# Precise identification of cascading alpha satellite higher order repeats (HORs) in T2T-CHM13 assembly of human chromosome 3

**DOI:** 10.3325/cmj.2024.65.209

**Published:** 2024-06

**Authors:** Matko Glunčić, Ines Vlahović, Marija Rosandić, Vladimir Paar

**Affiliations:** 1 Faculty of Science, University of Zagreb, Zagreb, Croatia; 2 Algebra University College, Zagreb, Croatia; 3 University Hospital Center Zagreb (Ret.), Zagreb, Croatia; 4 Croatian Academy of Sciences and Arts, Zagreb, Croatia

## Abstract

**Aim:**

To precisely identify and analyze alpha-satellite higher-order repeats (HORs) in T2T-CHM13 assembly of human chromosome 3.

**Methods:**

From the recently sequenced complete T2T-CHM13 assembly of human chromosome 3, the precise alpha satellite HOR structure was computed by using the novel high-precision GRM2023 algorithm with global repeat map (GRM) and monomer distance (MD) diagrams.

**Results:**

The major alpha satellite HOR array in chromosome 3 revealed a novel cascading HOR, housing 17mer HOR copies with subfragments of periods 15 and 2. Within each row in the cascading HOR, the monomers were of different types, but different rows within the same cascading 17mer HOR contained more than one monomer of the same type. Each canonical 17mer HOR copy comprised 17 monomers belonging to 16 different monomer types. Another pronounced 10mer HOR array was of the regular Willard's type.

**Conclusion:**

Our findings emphasize the complexity within the chromosome 3 centromere as well as deviations from expected highly regular patterns.

Recent dramatic advances in long-read sequencing, coupled with innovations in reading length and accuracy, have facilitated the generation of complete human chromosome assemblies such as T2T-CHM13 and have covered previously elusive complex structural variants (1-7). Until recently, the centromeric region of the human genome remained largely uncharted, resembling a genomic “black hole,” which restricted our ability to study the organization, variation, and function of centromeres. However, recent technological advancements have made it feasible to comprehensively investigate the structure and function of the complete human genome. The rich genetic variation concealed within these formerly inaccessible regions may have implications for both health and disease. In particular, these advances have spurred studies focusing on higher-order repeats (HORs). The unexplored variation underscores the necessity for more comprehensive T2T human genome assemblies derived from genetically diverse individuals. Altemose et al (4) initially identified certain HORs within complete genomic sequences characterizing the human centromeric region by employing a computational method previously introduced by Paar et al (8).

By studying the very limited sequencing data available in the past, it was discovered over a century ago that human centromeres contain approximately 171-bp alpha satellite repeat monomers, organized into sequences of *n* monomers, referred to as *n*mer HORs (9-22). Any two monomers within each HOR copy diverge from ~ 20% to 40%. However, HOR copies appear in tandem, with the divergence between HOR copies usually being less than 5%. Monomers exhibiting less than 5% of mutual divergence belong to the same monomer type. Willard et al found that, within each HOR copy, all constituent monomers belong to different monomer types. This pattern, known as Willard's type HORs, has been extensively studied using the limited sequencing data previously available, despite large gaps in the centromeric region (23-35).

In Willard's type *n*mer HOR arrays, the most common HOR copy with *n* constituting monomers is referred to as canonical. Copies in the same HOR array that contain inserts or deletions with respect to the canonical HOR copy are known as variants. The identification of HORs within a given genomic sequence presents a highly intricate computational challenge, requiring sensitive approximations. Until recently, this task was hampered by significant limitations in sequencing technology. The global repeat map (GRM) algorithm is a unique algorithm for precise identification of detailed HOR copies, both canonical and all its variants for the Willard՚s type of HORs (8,18,28,36-49).

There are various algorithms available for identifying higher-order periodicities within a given genomic sequence (50-58), owing to the computational complexity of the problem. The GRM algorithm offers a distinct advantage in enabling precise determination of HORs, facilitating the complete identification of both the length and structure of all HOR copies. This was recognized by Altemose (4), who used the algorithm NTRprism, which is similar to the GRM method from the study by Paar et al (8). However, one limitation of this approach is its design specificity for Willard's type HORs, characterized by only one monomer of each type in canonical HOR copies.

To address this limitation, we implemented a novel algorithm termed GRM2023, which represents an enhanced iteration of our prior GRM algorithm (8,18,28). GRM2023 extends its characterization beyond Willard's type HORs, further focusing on HORs with repeated monomer types within the canonical HOR copy. We termed these extended HORs as cascading higher-order repeats.

Providing a rigorous description of the structural organization of alpha satellite HORs poses a complex challenge, and discrepancies may arise between the results obtained with different methodologies. One notable advantage of the GRM and GRM2023 tools over alternative algorithms lies in their ability to achieve high precision in identifying HOR copies and elucidating their structure. GRM2023 detects peaks corresponding to alpha satellite HORs, as well as additional peaks that represent repeats (subfragments) not arranged in a tandem fashion. By using the GRM2023 algorithm, we were able to verify whether these additional peaks indeed corresponded to tandem repeats, thus enhancing the accuracy of our analyses.

Recent searches for the list of alpha satellite HORs within the complete T2T-CHM13 genome assembly of human chromosome 3 have yielded varying results, without precise identification of HOR copies. Previous findings (4) identified 17mer, 10mer, 5mer, and 4mer HORs, but also 17mer, 15mer, and 2mer HORs (4). In contrast, an earlier Southern blot analysis of human chromosome 3 (4) identified two primary Hind III fragments measuring 2.75 kb and 2.4 kb, which co-segregated in different human-hamster cell hybrids. These fragments corresponded approximately to ~ 16mer and ~ 14mer HORs, respectively. Additionally, a 650 bp fragment ( ~ 4mer HOR) was cloned and found to exhibit high specificity for the chromosome 3 centromere.

In this study, we precisely identified and analyzed alpha-satellite HORs using our high-precision GRM2023 algorithm applied to the complete T2T-CHM13 assembly of human chromosome 3.

## Methods

### GRM 2023 algorithm

The alpha satellite HORs were identified in the human chromosome 3 T2T-CHM13 genomic assembly by using the GRM2023 algorithm (18,41,42). The GRM2023 algorithm is specifically designed to detect and analyze very large repeat units, such as HORs, within genomic sequences. It generates a global repeat map in a GRM diagram, determining all prominent repeats in a particular sequence without any prior knowledge of the repeats. Once the consensus repeat unit is determined, it can be further combined with a search for dispersed HOR copies or individual constituting monomers.

For this study, we used two primary tools from the GRM2023 algorithm: MonFinder and GRMhor (both freely available at https://github.com/gluncic/GRM2023). In the first step, using the MonFinder application, we identified all alpha satellites in the human chromosome 3 T2T-CHM13 genomic assembly. The MonFinder tool takes genomic sequences (subject) and a consensus sequence (query) as input and delivers a list of detected monomers. This algorithm utilizes the Edlib open-source library for precise pairwise sequence alignment (59). Within the MonFinder algorithm, the subject sequence is searched in both the direct and reverse complement directions to identify all monomers. In this study, a unique consensus sequence of 171 base pairs (bp) in length, derived from over 1 000 000 different alpha satellites across all higher primates, including humans, was used as a query for detecting all alpha satellites in the T2T-CHM13 genomic sequence of human chromosome 3.

**Figure 1:**
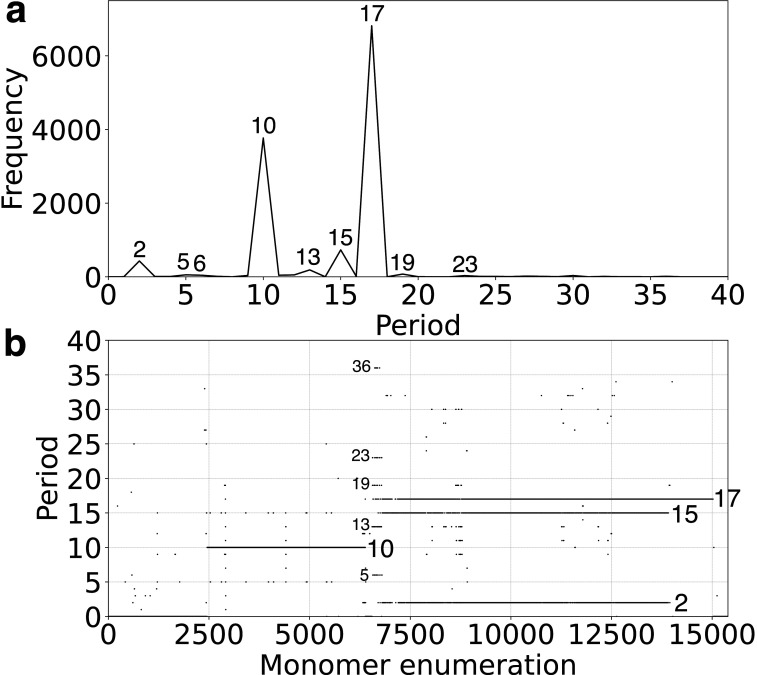
Global repeat map (GRM) diagram and monomer distance (MD) diagram for tandemly arranged alpha satellite monomers in complete T2T-CHM13 assembly of human chromosome 3. (**A**) GRM diagram. Horizontal axis: GRM periods. Vertical axis: the frequency of monomer repeats period. Identified major GRM peaks have periods 17, 15, 2, and 10, and minor peaks 15, 2, 13, 19, 5, 6. The significance of these GRM peaks (HORs or subfragment repeats) can be inferred from the MD diagram. (**B**) MD diagram. Horizontal axis: enumeration of tandemly organized alpha satellite monomers in order of appearance in GRM analysis of T2T assembly. Vertical axis: period (distance between the start of a monomer and of the next monomer of the same type). Two pronounced distinct regions with MD-line segments correspond to 17mer HOR (referred to as hor1) and 10mer (referred to as hor2). The additional MD-line segments at periods 15 and 2 correspond to subsegments of 17mer HOR. There also some additional weak repeats and sporadic MD points.

In the next step, the GRMhor application was executed with a file containing all alpha satellites from the previous step. The GRMhor application compares all the alpha satellites with each other and creates a divergence matrix. From the divergence matrix, monomer families were identified, encompassing all monomers that differ from each other by less than 5%. For each monomer family, a consensus sequence was generated. The consensus sequences for all alpha satellite monomer families are provided in Supplemental Table 1(Supplementary Table 1) and Supplemental Table 2(Supplementary Table 2). Furthermore, the GRMhor application generates a GRM diagram (Figure 1A), an MD diagram (Figure 1B), and an aligned schematic representation of the monomer organization in the array of monomers (Supplemental Figure 1(Supplementary Figure 1) and Supplemental Figure 2(Supplementary Figure 2)).

The GRM diagram displays the repetition period of alpha satellite monomers on the horizontal axis and the frequency of alpha satellite repeats period on the vertical axis. When generating the divergence matrix, the GRM algorithm assigns each alpha satellite its closest pair differing by less than 5%. The distance between two similar alpha satellites in the number of alpha satellites represents the repetition period. In this way, HORs and other alpha satellite repetitions are discerned as peaks in the GRM diagram. A distinct peak of period *n* (in units of 171 bp) represents an *n*mer HOR. Moreover, the GRM2023 algorithm facilitates the identification of various other types of monomer repeats, including intra- and inter-HOR-copy monomer repeats or tertiary HOR repeats, referred to as subfragments.

The monomer distance (MD) diagram displays the relationship between period and monomer enumeration (Figure 1B). Each point on the diagram represents a monomer enumeration on the horizontal axis and its corresponding distance to the next monomer of the same type in a sequentially organized monomer sequence, determining both horizontal and vertical coordinates. These points, termed MD points, form densely distributed horizontal MD-line segments corresponding to a HOR, with the vertical coordinate reflecting the period of the HOR. For a HOR, these MD-points are densely distributed on the line segment, and with the naked eye, they resemble a continuous line in the interval corresponding to constituting monomers. The top MD-line segment within an interval of monomer enumeration corresponds to the *n*mer HOR array, where *n* represents the period.

The NTRprism code (4) corresponds to the early version of the GRM code, and the NTRprism spectrum corresponds to the GRM diagram (18,41,42)). In the updated version of GRM used here, the GRM2023 code is extended to also identify the cascading HORs and interspersed HORs.

## Results and discussion

### GRM diagram

In the first step, we identified tandemly organized alpha satellite monomers in T2T-CHM13 assembly of human chromosome 3, enumerated in order of appearance in genomic assembly. Using the high-precision GRM2023 algorithm, we calculated the corresponding GRM diagram for this array of tandemly organized monomers. In this process, HORs were recognized as prominent peaks in the GRM diagram (Figure 1A). A peak of period *n* corresponds to *n ×* 171 bp, representing the *n*mer HOR. The most prominent GRM peaks for T2T-CHM13 assembly of human chromosome 3 corresponded to 17mer and 10mer HORs, with approximate frequencies of GRM peaks at ~ 7000 and ~ 4000, respectively.

The GRM2023 algorithm represents a novel iteration of the GRM algorithm, previously utilized for the identification of Willard's type HORs, characterized by the absence of repeat monomer types within a single HOR copy (12,18,20,37,48). In contrast, the GRM2023 algorithm is adept at discerning not only Willard's type HORs but also extends its capability to identify HORs exhibiting multiple occurrences of the same monomer type within a single HOR copy. These particular HOR instances are referred to as cascading HOR copies. Furthermore, the GRM2023 algorithm facilitates the identification of various other types of monomer repeats, such as intra- and inter-HOR-copy monomer repeats or tertiary HOR repeats, which are referred to as subfragments. In the case of T2T-CHM13 assembly of human chromosome 3, notable repeats of subfragment types were observed at periods 15, 2, 13, and 19, albeit with frequencies an order of magnitude lower than the two predominant peaks at 17 and 10.

### MD diagram

**Figure 2:**
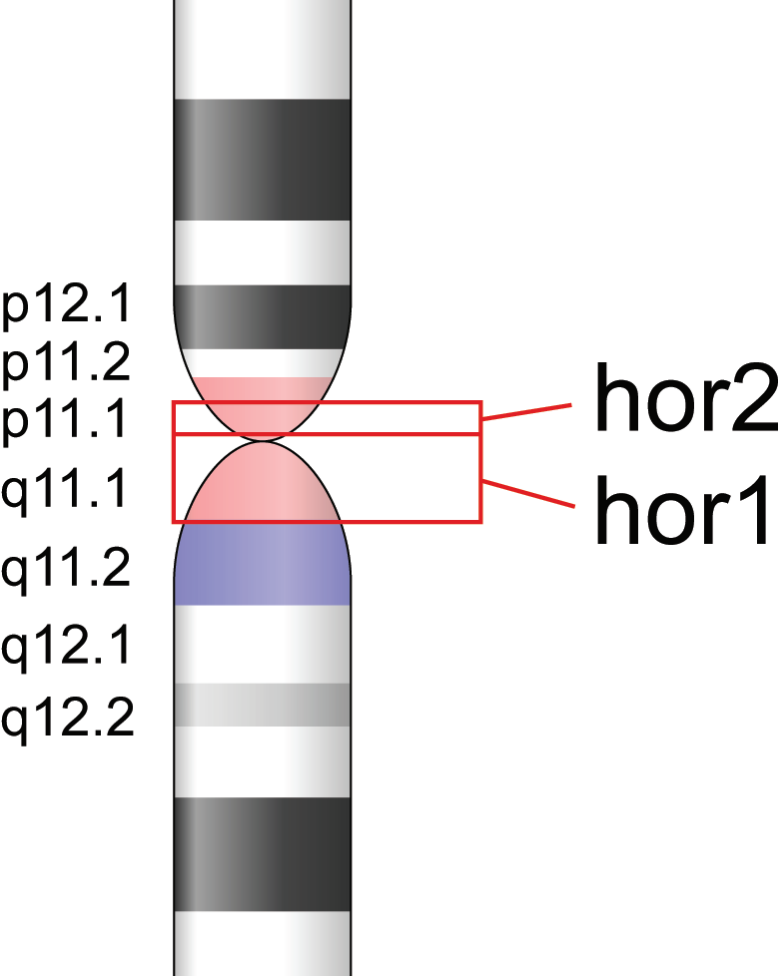
The ideogram of major alpha satellite higher-order repeats (HOR) arrays in the centromeric region of T2T-CHM13 assembly of human chromosome 3. hor1 – cascading 17mer HOR array; hor2 – Willard՚s type 10mer HOR array.

As seen from MD diagram (Figure 1B and Table 1), the most prominent MD-line segment corresponded to 17mer HOR. In the case of cascading HORs, additional parallel MD-line segments within the same interval of monomer enumeration may appear, exhibiting periods smaller than that of the 17mer HOR (subfragments). As seen from the MD diagram (Figure 1B and Table 1), in the case of 17mer HOR, the GRM peaks of periods 15 and 2 corresponded to subfragments. The sizable MD-line segments of different periods corresponded to the identified GRM peaks (Figure 1A): major peaks 17 and 10, and less pronounced weak peaks 15, 2 13, 19, 5, etc. The location of the 17mer and 10mer major HORs on chromosome 3 is shown in an ideogram (Figure 2).

**Table 1:** The frequency of monomer distance (MD) points for different periods. The number of MD points for two most frequent periods, 17 and 10, corresponds to the MD-line segments of two major HOR arrays: cascading 17mer and Willard-type 10mer HOR arrays, respectively. The periods 15 and 2 correspond to subfragments of 17mer HOR

No. of MD points	Period	Repeat pattern
6817	17	Cascading 17mer HOR
3679	10	Willard’s type 10mer HOR
731	15	subfragment of cascading 17mer HOR
430	2	subfragment of cascading 17mer HOR
188	13	subfragment*
74	19	subfragment*
54	5	subfragment*
52	12	
43	6	
43	11	
36	9	
34	30	
33	23	subfragment*
19	27	
15	16	
14	36	subfragment*

*subfragment denotes relation to a complex repeat in interval of monomer enumeration ~ 6500-6800 as mentioned in the text. The remaining less frequent periods correspond to other less pronounced repeats.

### Aligned scheme for cascading 17mer HOR array with 15mer and 2mer subfragments

**Figure 3:**
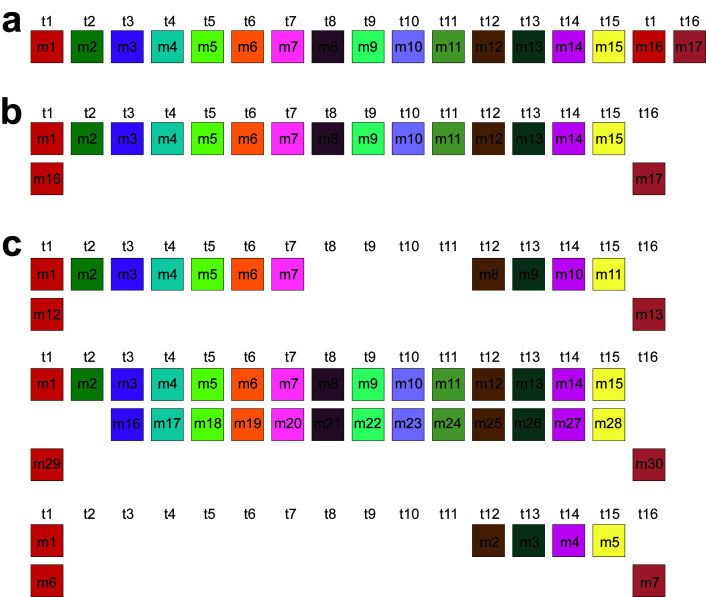
An aligned scheme of cascading 17mer canonical higher-order repeats (HOR) copies and some variants. (**A**) 17mer canonical HOR copy constituted of 17 monomers (denoted m1,… m17) of 16 different types (t1,… t16) presented in the linear monomeric scheme. The number of different types of monomers in the canonical HOR copy is denoted by τ. Each monomer is presented by a colored box. **(B)** A cascading aligned scheme of the canonical 17mer HOR (n = 17, τ = 16) corresponding to the linearized scheme in Figure 3A. Two monomers of the same type are aligned in the first column: monomer m1 of the type t1 in the first row and monomer m16 of the same type t1 in the second row. (**C**) Several examples of variant cascading HOR copies from Supplemental Figure 1(Supplementary Figure 1): 13mer, 30mer, and 7mer with respect to 17mer HOR array.

As inferred from the GRM and MD diagrams (Figure 1 A,B), the largest array of HORs within human chromosome 3 is identified as the cascading 17mer HOR, spanning the genomic interval from 91 779 888 bp to 96 415 046 bp in the T2T-CHM13 assembly. The comprehensive alignment pattern of the cascading 17mer HOR array, computed using the GRM2023 algorithm, is depicted in Supplemental Figure 1(Supplementary Figure 1). Additionally, the predominant constituent of this array, namely the canonical 17mer HOR, is depicted through a linear arrangement of its constituent 17 monomers (Figure 3A).

Monomers within the 17mer HOR copy, labeled m1 through m17 in order of appearance within the canonical HOR copy, are arranged sequentially in a linear fashion, each represented by a distinct colored box. Above each box stands its corresponding type, labeled as t1, t2, and so forth. Different monomer types are distinguished by varying box colors, while monomers of the same type share identical coloring.

The two 17mer cascading HOR monomers, m1 and m16, are classified under the same type, denoted as t1. In the instances where the canonical copy exhibits a repetition of monomer types, the linear presentation of the HOR copy is transformed into a cascading format, resulting in a multi-row arrangement. Each row consists of monomers of distinct types, aligned vertically according to their respective types.

Consequently, the linear single-row depiction of the 17mer canonical HOR copy (Figure 3A) is transformed into a two-row representation as depicted in Figure 3B. The first row comprises a linear sequence of monomers, m1 through m15, corresponding to types t1 through t15, respectively. The second row features only two monomers: m16, type t1, aligned with m1 of the same type from the first row, and m17, designated as type t16, positioned to the right of m15 in the first row. This presentation, characterized by aligned monomers based on their types, is termed cascading 17mer HOR (Figure 3B).

**Figure 4:**
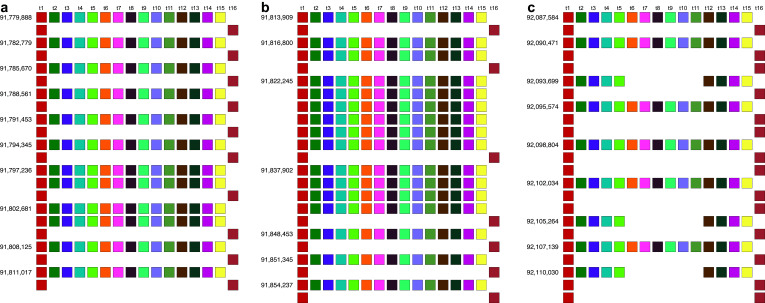
An aligned scheme of some segments from cascading 17mer higher-order repeat (HOR) array. (**A**) A segment of the first ten cascading 17mer HOR copies from position 91 779 888 to 91 811 017. Each HOR copy corresponds to cascading rows of monomers. The No.1 HOR copy is canonical, consisting of two cascading rows: the first row with 15 monomers of types t1-t15 and the second row with two monomers of types t1 and t16. The next five HOR copies, No. 2-6, are of the same canonical structure. The cascading HOR copy No. 7, starting at position 91 797 236, is a variant HOR consisting of 15 + 15 + 2 = 32 monomers (three cascading rows of 15, 15, and 2 monomers, respectively). This variant HOR copy arises from the canonical HOR copy by duplicating its first row. The next HOR copy, No.8, starting at position 91 802 681 is the same as HOR copy No. 7. The next two HOR copies, No.9 and 10, are canonical (15 + 2). (**B**) A segment of cascading 17mer HOR copies from position 91 813 909 to 91 854 237. This segment starts with canonical 17mer HOR copy (15 + 2 monomers). The following copy is an extended HOR copy of 2 × 15 + 2 = 32 monomers, which arises from the canonical 17mer HOR copy by multiplication of the first row in the canonical HOR copy. The next copy is an extended HOR copy of 6 × 15 + 2 = 92 monomers, which arises from the canonical 17mer HOR copy by multiple multiplication of the first row in the canonical HOR copy. The next copy is a variant HOR copy of 4 × 15 + 2 = 62 monomers, which arises from the canonical 17mer HOR copy by multiplication of the first row in the canonical HOR copy. After that follows a sequence of canonical 17mer HOR copies. (**C**) A segment of cascading 17mer HOR copies from position 92 087 584 to 92 110 030, giving rise to a tertiary period-2 subfragment. This graphical presentation is also presented in Table 3. The sub-tandem of (t1, t16) doublets within HOR copies gives rise to subrepeats … t2 t15 t2 t15 …, which due to distances t2-t2 and t15-t15 of 2 × 171 bp generates intra-HOR tertiary periodicity 2.

Variants involving adjacent rows, such as (t1, t16) and (t1, t16), exemplified by the 30mer variant in Figure 3C, also contribute to the subfragments of period 2 as a consequence of tertiary HOR. Select segments of the array of cascading 17mer HOR copies from Supplemental Figure 1(Supplementary Figure 1) are depicted in Figure 4.

It is possible to inspect the accompanying subfragments considering the types of monomers in the canonical 17mer HOR copy. The 17 monomers, m1 m2 m3 m4 m5 m6 m7 m8 m9 m10 m11 m12 m13, m14 m15 m16 m17, in the canonical 17mer HOR copy have the corresponding monomer types, t1 t2 t3 t4 t5 t6 t7 t8 t9 t10 t11 t12 t13 t14 15 t1 t16, which for simplicity we write 1 2 3 4 5 6 7 8 9 10 11 12 13 14 15 1 16. Analogously, the monomer types in the corresponding neighboring canonical 17mer HOR copy are denoted 1՚ 2՚ 3՚ 4՚ 5՚ 6՚ 7՚ 8՚ 9՚ 10՚ 11՚ 12՚ 13՚ 14՚ 15՚ 1՚ 16՚. Let us consider the two neighboring canonical 17mer HOR copies:

**1** 2 3 4 5 6 7 8 9 10 11 12 13 14 15 **1** 16 **1՚** 2՚ 3՚ 4՚ 5՚ 6՚ 7՚ 8՚ 9՚ 10՚ 11՚ 12՚ 13՚ 14՚ 15՚ **1՚** 16՚.

Within the first HOR copy, the distance *d* between the start of two monomers of identical type t1 between the monomer denoted m1 and the monomer denoted m16 in the initial m-sequence is equal to 15 units of monomer lengths, ie, equal to the sum of lengths of monomers m1, m2, … m15, *d* = 15. This is the characteristic intra-HOR-copy distance within each 17mer canonical HOR copy and it gives rise to the MD-line segment of period 15 in the MD diagram. It is referred to as a period-15 subfragment. For tandems of canonical 17mer HORs, this pattern is equidistant.

Furthermore, the inter-HOR-copy-distance between the second monomer of type 1 in the first HOR copy and the first monomer of type 1 (denoted 1՚) in the second HOR copy:

… 15 **1** 16 **1՚** 2՚ …

is equal to the sum of lengths of monomers of type 1 and of type 16, *d* = 2. Based on this principle, we obtained two MD-line segments at periods 15 and 2, referred to as subsegments. They are positioned in the same interval of monomer enumeration as the line segment corresponding to the 17mer HOR (Figure 1B). The onset of period 2 arises also due to contributions from variants of 17mer HOR copy, involving tandem repeats of t1 t16 doublets within HOR copies (Figure 4C). A segment of the cascading 17mer HOR contributing to period 2 repeats is provided in Supplemental Table 2(Supplementary Table 2) (Table 2).

**Table 2:** A segment from Supplemental Table 2(Supplementary Table 2) of cascading 17mer higher-order repeats (HOR) contributing to period 2 repeats

Monomer type	Repeat pattern
t1-t15	Variant15 + 2 + 2
t1, t16	
t1, t16	
t1-t 5, t12-t15	Variant (6 + 4)+2
t1, t16	
t1-t15	Variant15 + 2 + 2
t1, t6	
t1, t6	
t1-t15	Variant15 + 2 + 2
t1, t6	
t1, t6	
t1-t15	Variant15 + 2 + 2
t1, t6	
t1, t6	
t1-t6, t12-t15	Variant (6 + 4)+2
t1, t16	
t1-t15	Canonical 15 + 2
t1, t16	
t1-t5, t12-t15	Variant (6 + 4)+2 + 2
t1, t16	
t1, t16	
t1-t5, t12-t15	Variant (6 + 4)+2 + 2
t1, t16	
t1, t16	
t1-t5, t12-t15	Variant (6 + 4)+2 + 2
t1, t16	
t1, t16	
t1-t15	Canonical 15 + 2
	
t1, t16	

Furthermore, within the specific range of monomer enumeration spanning from ~ 6500 to ~ 6800, a highly intricate repeating pattern emerged, comprised of subfragments with periods of 23, 19, 17, 13, 6, and occasionally a less pronounced 36.

**Figure 5:**
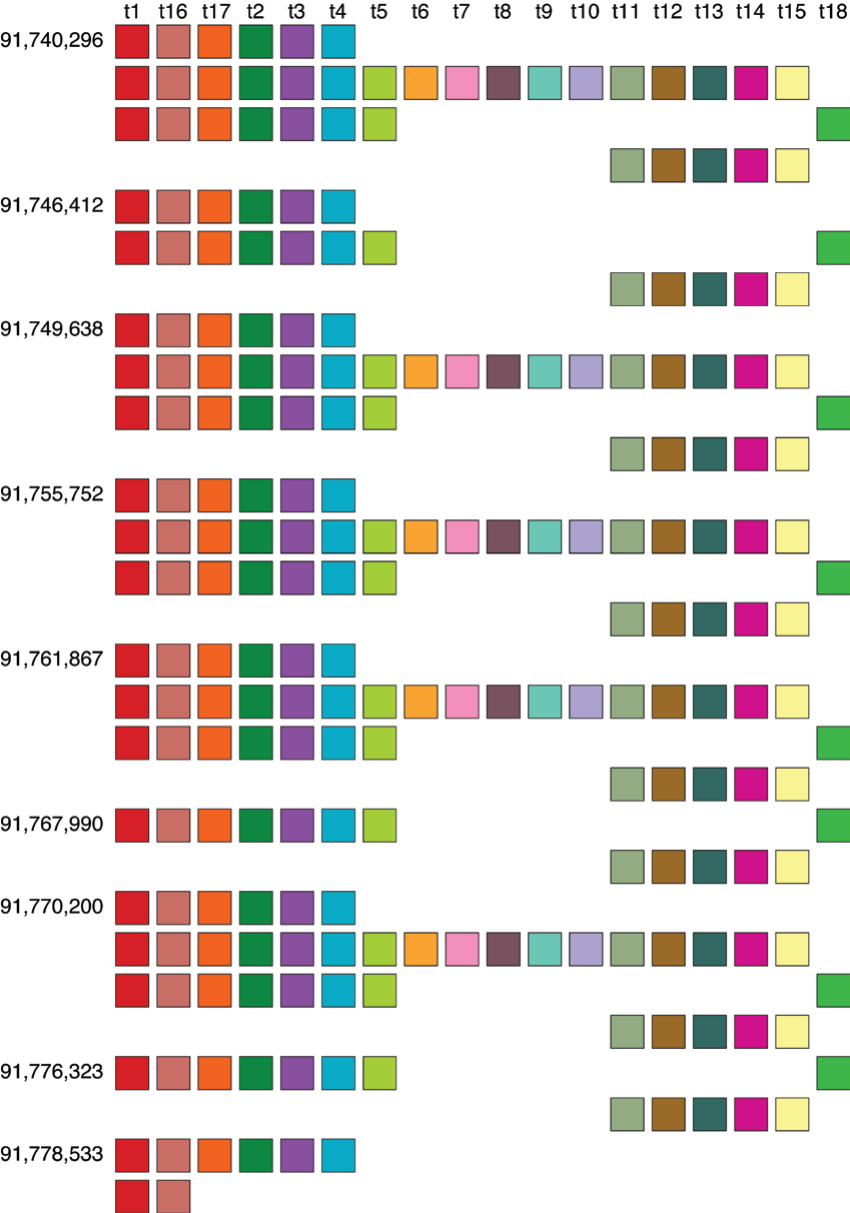
An aligned scheme of the entire array of cascading 36mer alpha satellite higher-order repeat (HOR). The number on the left side indicates the initial position of the first monomer in each row of HOR copy. The box colors and monomer type labels align consistently with those of the 17mer HOR. There are five canonical copies of the cascade 36mer HOR, primarily composed of the same monomers found in the 17mer HOR. Each HOR copy corresponds to cascading rows of monomers. The HOR copies No. 1, 3, 4, 5 and 7 are canonical, consisting of four cascading rows: the first row with 6 monomers of types t1, t16, t17, t2-t4; the second row with 17 monomers of types t1, t16, t17, t2-t15; the third row with 8 monomers of types t1, t16, t17, t2-t5, t18; and the forth row with 5 monomers of types t11-t15 (6 + 17 + 8+5 = 36). Between these canonical HOR copies, four-variant HOR copies with significantly fewer monomers in each HOR unit are dispersed.

Figure 5 illustrates all HORs in this region, with box colors and monomer type labels consistent with those of the 17mer HOR shown in Figures 3 and 4. There exist five canonical copies of the cascade 36mer HOR, predominantly composed of the same monomers as the 17mer HOR (Figure 5). Each canonical 36mer HOR includes 16 distinct types of monomers present in the 17mer HOR (t1 to t16) along with two additional monomer types, t17 and t18. These monomers are largely arranged in the canonical 36mer HOR in the same sequence as in the canonical 17mer HOR, except for the insertion of monomers t16 and t17 between t1 and t2. Furthermore, the canonical 36mer HOR is characterized by a significant number of monomer duplications, with each individual HOR unit containing three copies of t1, t2, t3, t4, t16, and t17, as well as two copies of t5, t11, t12, t13, t14, and t15. Thus, from only 18 distinct monomer types, a 36mer HOR is formed, resulting in a large number of subfragments in Figure 1B. Following the final variant copy of this HOR, commencing at position 91 778 533, the 17mer HOR continues.

### Aligned scheme for Willard՚s type alpha satellite 10mer HOR array

**Figure 6:**
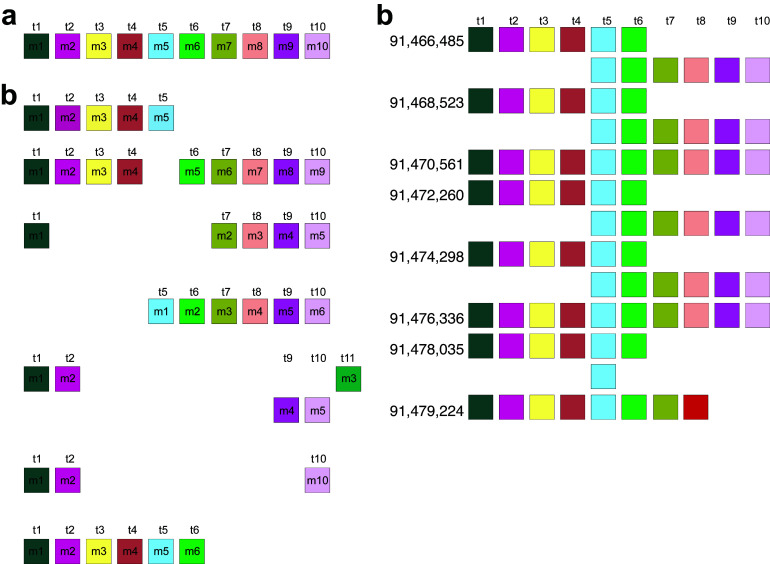
An aligned scheme of some segments from Willard՚s type 10mer HOR array. (**A**) a scheme of canonical 10mer HOR copy. (**B**) A scheme of several variants in 10mer HOR array. (**C**) A cluster of canonical and variant HOR copies (2 canonical and 11 variant) at the end of 10mer HOR array.

As observed in the MD diagram (Figure 1B), the 10mer HOR array, designated as hor2, is situated within the monomer enumeration interval between ~ 2500 and ~ 6400 determined from T2T-CHM13 assembly. The aligned 10mer HOR scheme for this 10mer HOR array is presented in Supplemental Figure 2(Supplementary Figure 2), and the consensus HOR is displayed in Supplemental Table 2(Supplementary Table 2). Specific segments from the aligned 10mer HOR scheme are shown in Figure 6. The composition of HOR copies in 10mer HOR array from Supplemental Figure 2(Supplementary Figure 2) is analyzed in Supplemental Table 3(Supplementary Table 3).

The high percentage of copies (94%) were canonical HOR copies. Variant HOR copies showed a strong tendency of clustering in large groups of 76%, 55%, 50%, 50%, and 47%, scattered between large groups of canonical HOR copies, but the composition of monomer types in variants was far from random. Among the monomer types, the most frequent in variants were t-t5, t-t6, and t5-t10. Half of the variants were located near the end of 10mer HOR array, as transitional region in dissolving the HOR regularity.

In conclusion, by using the recently sequenced complete T2T-CHM13 assembly of human chromosome 3, we delineated the precise alpha satellite cascading HOR structure by employing our innovative high-precision GRM2023 algorithm with GRM and MD diagrams. This study rigorously identified and structurally analyzed alpha satellite HORs within the centromere. Notably, the major alpha satellite HOR array in chromosome 3 revealed the novel cascading 17mer HOR.
