# Supplementary material for: Precise identification of cascading alpha satellite higher order repeats (HORs) in T2T-CHM13 assembly of human chromosome 3
Source: Croat Med J. 2024 Jun;65(3):209–20. doi: 10.3325/cmj.2024.65.209 (PMC11157248; doi:10.3325/cmj.2024.65.209)
Supplement: Supplementary Table 1 [file CroatMedJ_65_s001.pdf]

Table S1. Canonical 17mer consensus sequence.

Monomer type t1
TCAGTAGCTTCTTTGTGTGTGTGTATTCAACTCACAGAGTTGAACCTTCCTTTAGACAGAGCAG
ATTGGAAACACTCTTTTTGTGGAATTTGCAAGTGGAAAATTCTAGCAGTATGAGGCCAATGGTA
CAAAAGGAAATATCTTCGTATAAAAACTAGACAGTATCATTC
Monomer type t2
TCAGGAACTACTTTGTGATATGTGCATTCAACTCACAGAGTTTAACCTTTCTTTTCATAGATGA
GTTTGGAAACAGTCAGTTTGTAAATTCTGCAACTGGATATTTGGACCTCTTTGAGGCTTTCGTT
GGAAACGGGATTTCTTCACATAATGCTAGACAGAAGAATTC
Monomer type t3
TCAGTGAATTTTTTTCTGTGTGTGTGTATTCAACTCACAGGGTTGAACCTTCCTTTAGACAGTG
CAGATTTGAAACACTTGTCTGTGGAATTTGCAAGGGGAGATTTCAAGCACTTTGAGGCCATTGG
TGGAAAAGGAAATATCTTCGTATAAAAACTAGACAGAATCATTC
Monomer type t4
TCAGAAACTGCTTTGGGATGTGTGCATTGAACTCACAGTGTTTAACACTTCTTTTCATAGAGCA
CTTTGGAAACACTCAGTTTGTAATGTCTGCAGCTGGATATTTGGACCTCTTTGAGGCCTTCGTA
GTAAACGGGATTTCTTCGTGTAATGATAGACAATAGAATTC
Monomer type t5
TCAGTAACTTCTTTTTGTGGTGTGTATTCAACTCACAGAGTTGAACCTTCCTTTAGACAGAGCA
GATTTGAAACTCTCTTTTTGTGGAATTTGCAAGTGGAGATTTCAAGCGCTTTGAGGCCAACGGC
AGAAAAGGAAATATCTTCGTAGAAAAAATAGACGGAATCATTC
Monomer type t6
TCAGAAACTACTTTGTGATGTTTGCGTTCAACTCACAGAGTTTAACGTTTCTTTTCATAGAGCA
GTTTGGAAACACTCTTTTTGCAGAATCTGCAAGTGGATATTTGGACCTCTTTGTGGCCTTCGTT
GGAAACGGGATTTTTCATATAATGCTAGACAGAAGAATTC
Monomer type t7
TCAGTAACTTCTTTGGGTTGTGGGTATTCAACTCACAGAGTTGAAGCTTCCTTTAGGCGGAGCA
GATTGGAAACACTTTTTGTGGAATTTTCAGGGGGAGACTTCAAGCGCTTTGAAGTGAATGGTAG
GAAAGGAAATATCTTCGTATAAAAACTAGACGGAGTCATTC
Monomer type t8
TCAGAAACTACTTTGGTACGTGTGTGTTCAACTCACAGTGTTTAACCTTTCTTTTCATAGAGCA
GTTTGGAAACACTCAGTTTGTAAAGTCAGCAACTGGATATTTGGATGTATTTGAGGCCTTCGTT
GGAAACGGGATTTCTTCATATAATGCTAGACAGAAGAATTC
Monomer type t9
TCAGTAACTGCTTTTTCTGGTGTGTATTCAACTCTCAGAGTTGAACTTTCCTTTAGAAACAGCA
GATTTGAAACTCTCTTTTTGTGGAATTTGCAAGTGGAGATTTCAGAGCTTTGAGGCCAATGGTA
GAAAAGGAAATATCTTCGTATGCAAACTAGACAGAATCATTC
Monomer type t10
TCAGAAACTGCTTTGCAATGTGTGCGTTCAACTCACAGTGTTTAACCTTTCTTTTCATACAGTT
GTTTCGAAACACTCTTTTTGCAGAATCTGCAAGTGGATATTTGGACCTCTTTGAAGTCTTCGTT
GGAAATGGGATTTCTTCATATAATGCTAGACAGAAGACTTC
Monomer type t11
TCAGTGAATTCTTTCTGTGTGTGTGTATTCAACTCACAGAGTTGAACGTTCCTTTAGACAGAGT
AGATTGGAAACACTCTTTTTGTGGAATTTTCAGGTGGAGGTATCAAGCGCTTTGAGGCCAATGA
TAGAAAAGGAAATACCTTCGTATAATAATTAGACGGAATCATTC
Monomer type t12

TCAGAAACTGAGTTGTGATGTTTGCATTCAACTCACAGAGTTCAACATTCCTTTTAATGGAGCG
GTTTTGAAACACTCTTTTTGCAGAATCTGCAAGTGGATATTTGGACCTCTTTGAGGCCTTCGTT
GGAAACGGGATTTCTTCATGTAATGCCAGACAGAAGAATTC

Monomer type t13

GCAGAAACCACGTTGTGATCTCTGCATTCAACTCACAGAGTTGAACCTTTCTTCCTATAGAGCA
GTTATGAAACAGTCTCTTTGTAGAATTTGCAAGGGTGTATTTAGAGGGCATTGAAGCCTACGGT
AGAAAAGGAAATATCTTACCATAAAATCTAGTCAGAAGCATTC

Monomer type t14

TCAGAAACTTCTTGATTGTTATGTGTGCATTCAACTCACAGAGTTGAACCTTACTTTGGAAAGA
GCAGTTTTCTAACACTCTTTTTGTAAAAGTTCCAAGTGAATACTTTGAGTGCTTTGAAGCCTAC
GGTTGACAACGAAATATCTTCATGTAAAAACTACAAAGAATCATTC

Monomer type t15

TCAGAAACTACTTTGTGATGTGTGCGTTCAACTCACAGAGTTTAACCTTTCTTTTCATAGAGCA
GTTTGGAAACACTCTGTTTGTGAAGTCTGCAAGTGGATATTTAAACGTCTTTGAGGCCTTCGTT
GGAAACGGGATTTCTTCATATAAACCAGGACAGAAGAATTC

Monomer type t16

TCAGAAACTGCTTTGTGATGTGTGTATTAAACTCACAGAGTTGAACATTTCTTTGCATAGAGCA
GTTTGGAAAGACTTAGTTTGTGCAGTGTGCAAGTGGATATTTGGAACTCTTTGAGGCCTTCGTT
GGAAACGGGATTTCTTCTTATAATTCTTGACAAAAGAATTC
