# Supplementary material for: Precise identification of cascading alpha satellite higher order repeats (HORs) in T2T-CHM13 assembly of human chromosome 3
Source: Croat Med J. 2024 Jun;65(3):209–20. doi: 10.3325/cmj.2024.65.209 (PMC11157248; doi:10.3325/cmj.2024.65.209)
Supplement: Supplementary Table 2 [file CroatMedJ_65_s002.pdf]

Table S2. Canonical 10mer consensus sequence.

Monomer type t1
TCATAAACTTCTTCGTGATGTGTGCTTTCAACTCGCAGCGTTGAAGCTTCCTTTCGATAGAGCA
GTTTTGTAACTCTCTTTTTGTAGAATTTCCAAGTGGATATTTAGCGCCGTTTGAGGCCTATGGT
GGAAAAGGCAATATCTTCATAGAAAAACTAGACAGAATGATTC
Monomer type t2
TCGGAAACTACTTTGTGATACCTGCCTTCAACTCTCAGAGTTGAATATTCCTCTTGATGGAGCA
GTTTTGAAAAACTCTTTTTGTTGAATCTCCAAGTGGATATTTGGACCTCTTTGTGGCCTTCGTT
TGAAACGTGACTGCTTCATACAAAAGTAGACAGAAGAATTC
Monomer type t3
TCTGAAGCTACTTTGTGATGTGCGCATTCAGCTGACAGAGTTTAACCTTTCTTTGGATAGAGCG
GTTTTAAACACTCTTTTTGTGGAATTTGCAATTCTATATTTAGAGTGCTTTCAGGCCTGTGGTA
CAAAAGGGAATGTCTTCACATAAAATCTAGACAGAAGCATTG
Monomer type t4
TCAGAAACTTCTCTGTGATGTGTGCATTTAACTCTCAGAGTTCAACCTTCCTTTTGATAGAAGA
GTGTTGAAATATTCTTTTTGTAGAATTTCCAAGTGAATATTTAGAGCGGTTTCAGGCCTATGTA
GAAGAGAAAATATCTTCACAGAAAAACTAGACATAATTGTTC
Monomer type t5
TCAGAAACTACTTTGTGATGTGTGGGTTCAACTCACTGAGTTTAACCTTTCTTTTGATAGACCA
GTTATGAAACACTCTTTTTGTAGAATCTGCAAGTAAATATTTGGACTTTTTTGAGGCCTTCATT
GGAAACGGGATTTCTTCATATAAACCTTGACAGAAGAATTC
Monomer type t6
TCAGAAACTTCTTTGTGATGTGTACCTTCAACTCACAGAGTTGAAGCTTCCTTTCAATAGAGCA
CTTTTGAAACTCAGTTTTTGTAGAATTTCCAGGTGGATATTTAGCGCCGTTTGAGGCCTATGGT
AGAAAAGGCAATATCTTCGTAGGAAAACTAGACAGAATGATTC
Monomer type t7
TCAGAAACTTATTTGTGATATTTGCATTCAACGCACAGAGTTGAACATTCCTCTTGATGGAGCC
GTTTTGAAACACTCTTTTTGTAGAATCTGCAAGTGGATATTTGGACCTCTTTGTGGCCTTCGTT
TGAAACGTGATTTCTTCATTTACAACTAGACAGAAGAATTC
Monomer type t8
TCCGAAGCTGTTTTGTGATGCTTGCATTCAGCTGACAGAGTTTAAACTTCCTTTGATAGAGCAG
TTTGGAAACACTCTTTTTGTGGAATTTGCAAGTGTATATTTAGAGCGTTTTGAGGCCTACAGTA
GGAAAGGAAATATCTTCACATAAAAACTAGACAGAAGTATTG
Monomer type t9
TCAGAAAATTATTTGTGATATGTGCATTTAACTCATGGAGTTGAAACTTCCTTTCGATAGAAGA
GTTTTGAAATACTCTTTTTGTAGAATTTCCAAGTGGATTTTTACAGCGGTTTGAGGTCTATGGC
AGAAAAAGAAATATCTTCACAGAAAAACTAGGCAGATTCATTC
Monomer type t10
TCAGAAACTACTTTGTGATGTGTGCCTTCAACTCACAGAGTTTAACCTTTCTTTTGATAGAGCA
GTTTTGAAAAACTCTTTTTGTAGAATCTGCAAGTGTATATTGGGACTTTTCTGAGGCCATCTTT
GGAAACGGGATTTCTTCATATAAAACTTGAAAGAAGAATCC
