# Supplementary material for: Precise identification of cascading alpha satellite higher order repeats (HORs) in T2T-CHM13 assembly of human chromosome 3
Source: Croat Med J. 2024 Jun;65(3):209–20. doi: 10.3325/cmj.2024.65.209 (PMC11157248; doi:10.3325/cmj.2024.65.209)
Supplement: Supplementary Table 3 [file CroatMedJ_65_s003.pdf]

Table S3. HOR copy composition of 10mer HOR array. CHC, canonical HOR copy; var, variant HOR copy.

No. of HOR Copies	HOR copy
9	CHC
1	var t1-t5
27	CHC
1	var t1-t5
8	CHC
1	var t1-t4, t6-t10
1	CHC
2	var t1-t5
50	CHC
1	var t1-t5
55	CHC
1	var t1, t7-t10
12	CHC
1	var t6-t10
30	CHC
1	var t4-t10
1	var t3, t7-t10
50	CHC
1	var t1-t2, t11
1	var t9-t10
1	CHC
1	var t1-t2, t11
1	var t9-t10
47	CHC
1	var t1-t2, t10
1	var t9-t10
1	var t1-t2, t10
1	var t9-t10
1	var t6-t10
11	CHC
1	var t1-t6
1	var t2-t10
76	CHC
1	var t1-t6

1	var t5-t10
1	var t1-t6
1	var t5-t10
1	var t1-t6
1	var t5-t10
1	var t21-t6
1	var t5-t10
1	CHC
1	var t1-t6
1	var t5
1	var t1-t8
