# Supplementary material for: Precise identification of cascading alpha satellite higher order repeats (HORs) in T2T-CHM13 assembly of human chromosome 3
Source: Croat Med J. 2024 Jun;65(3):209–20. doi: 10.3325/cmj.2024.65.209 (PMC11157248; doi:10.3325/cmj.2024.65.209)

Fig. S1. Cascading 17mer alpha satellite HOR alignment. Start position 91,779,888 bp and end position 96,415,046 bp in T2T- CHM 13. The number on the left side indicate the initial position of the first monomer in each row of HOR copy.

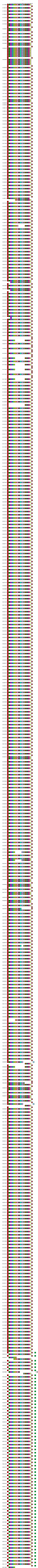

Supplement: Supplementary Figure 1 [file CroatMedJ_65_s004.pdf]
