# Supplementary material for: Precise identification of cascading alpha satellite higher order repeats (HORs) in T2T-CHM13 assembly of human chromosome 3
Source: Croat Med J. 2024 Jun;65(3):209–20. doi: 10.3325/cmj.2024.65.209 (PMC11157248; doi:10.3325/cmj.2024.65.209)

Fig. S2. Willard's type 10mer alpha alpha satellite HOR alignment. Start position 90,807,672 bp and end position 91.480,588 bp in T2T- CHM 13. The number on the left side indicate the initial position of the first monomer in each row of HOR copy.

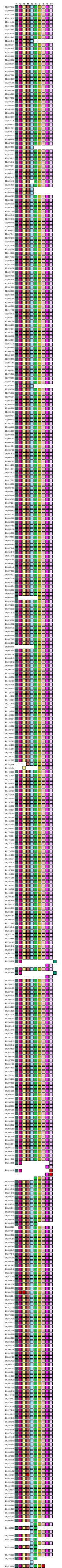

Supplement: Supplementary Figure 2 [file CroatMedJ_65_s005.pdf]
